# Arterial Blood Supply of the Stifle Joint in Horses

**DOI:** 10.3390/ani14091279

**Published:** 2024-04-24

**Authors:** Hanna Schöpper, Monika Egerbacher

**Affiliations:** 1 Institute of Topographic Anatomy, Department of Pathobiology, University of Veterinary Medicine, 1210 Vienna, Austria; 2 Institute of Anatomy, Anatomy and Cell Biology, Faculty of Medicine, University of Bonn, 53111 Bonn, Germany; 3 Institute of Anatomy, Neuroanatomy, Faculty of Medicine, University of Bonn, 53111 Bonn, Germany; 4 Institute of Pathology, Working Group Histology and Embryology, Department of Pathobiology, University of Veterinary Medicine, 1210 Vienna, Austria

**Keywords:** equine, femoropatellar joint, femorotibial joint, knee, vascularization, popliteal artery

## Abstract

**Simple Summary:**

In former times, horses with stifle injuries could not be helped much due to restrictions in diagnostic and therapeutic options. With the technical progress and willingness of owners to pay for advanced veterinary services, operations to the stifle joint became feasible. As a detailed anatomical description of the blood supply in the stifle region was lacking for horses, the aim of the study was to fill this gap. Vessels were dissected and described so that a detailed map for the blood supply of the horse stifle is now available. This information will help veterinary surgeons to minimize unwanted damage or bleeding during operations.

**Abstract:**

The vascularization pattern of the equine stifle joint is insufficiently described in the literature, even though there is a growing need for knowledge of the exact blood supply, as (i) arthroscopy and endoscopic surgery techniques are increasingly performed in horses and (ii) ex vivo models of menisci need nutrient supply that mimic the in vivo situation. The aim of this study was to describe the vessels involved in the stifle joint supply and the exact branching pattern of the popliteal artery. Colored latex was injected into the arteries of nine pelvic limbs of equine cadavers (n = 6) to evaluate the occurrences, variations and approximate diameters of vessels that supplied the stifle joints. Next to a branch of the saphenous and descending genicular arteries, eleven branches of the popliteal artery could be described in horses that feed the vascular network of the stifle joint. With a focus on the blood supply of the menisci, a vascularization map was created to show the main influx to these intra-articular structures in detail. These findings are potentially of great importance to both clinicians in preparation of best-suited incisions for arthroscopy and researchers designing new approaches for meniscal studies and choosing suitable animal models.

## 1. Introduction

The stifle joint of horses (*Equus caballus*) is a compound joint consisting of two articulations: the femoropatellar (FP) and femorotibial (FT) joints.

The bones joined at the FP joint are the femoral trochlea covered with cartilage and the sesamoid bone of the quadriceps femoris muscle, i.e., the patella. Para- and suprapatellar fibrocartilage ensures that the patella stays within the trochlear grove during maximal flexion. The articular cavity is enclosed by a roomy articular capsule, which is composed of an inner synovial membrane and an outer fibrous capsule. Ligaments that stabilize the stifle joint are the medial and lateral femoropatellar ligaments, patellar ligaments (intermediate, medial and lateral patellar ligaments), and medial and lateral additional ligamentous thickenings of the capsule [1,2]. The popliteal tendon runs intra-synovially and inserts at the lateral epicondyle of the femur. It is considered to stabilize the posterior-lateral region of the stifle joint [3].

For the FT joint, the femoral condyles interact with the proximal articular surface of the tibia via the menisci. The menisci are fibrocartilaginous structures that compensate the incongruity of proximal and distal articular components. The articular cavity is subdivided into a lateral and a medial compartment that may communicate with each other [4,5]. The FT joint is stabilized by two cruciate ligaments (cranial and caudal cruciate ligament), six meniscal ligaments and two collateral ligaments.

The stifle joint plays a crucial role in the locomotion of horses, where it is the main source of propulsive and jumping force from the pelvic limb. Despite the huge forces that act on the stifle, the FT joint in particular comprises not only large bones but also delicate structures, like the menisci and cruciate ligaments. These structures are especially vulnerable and at risk to be injured or to develop chronic pathologies, particularly in sport horses [6].

In the intensive training of sport horses, but also in elderly equine patients or after a severe trauma, pathologies of the stifle joint may occur and are more frequently diagnosed because of improved imaging techniques [7,8]. Since a classical surgical approach to the equine stifle is rather difficult, arthroscopic techniques are performed to an increasing extent [9,10]. The healing rates of such small incision approaches are better than ever, and thus, many owners nowadays tend to decide on this form of diagnostic and surgical approach.

Different arthroscopic techniques are used in daily equine veterinary practice [9,11,12]. The lateral and medial joint compartments of the FT joint are separated by the fascial sheath in the median intercondylar region where cruciate ligaments and popliteal arteries and veins are located. The lateral and medial joint compartments of the FT joint usually do not communicate [5]. Both are divided into a cranial and a caudal sac. The caudal lateral synovial sac is composed of two pouches, i.e., a proximal and a distal pouch, separated by the popliteal tendon. Communication between these two pouches exists [13]. The medial synovial joint compartment is in conjunction with the FP joint sac [4]. The arthroscopic approach of the cranial joint sacs is comparatively simple and is performed by inserting an arthroscope via a skin incision between the middle and medial femoropatellar ligament [9]. For the visualization of the caudal parts of the articular cavities, two different approaches caudal to the medial and lateral collateral ligaments are taken into consideration [14]. Furthermore, two cranial approaches to the caudal sacs are described: (i) via the intercondylar space underneath the caudal cruciate ligament [15] and (ii) via the popliteal tunnel into both pouches of the lateral caudal sac of the femorotibial joint [12].

All approaches to the FT joint have the risk of injuring either articular cartilage, menisci, ligaments, nerves or vessels. In the cranial approach to the caudal sac via the intercondylar space, the risk of injuring the popliteal artery is mentioned but described as minor due to the oblique positioning of the arthroscope [15]. In other publications, the risk of injuring the popliteal artery or its branches is not mentioned [12,16]. The underestimation of potential vascular trauma during surgery is presumably due to the lack of information about the vasculature in this region. For equine surgeons, the lack of detailed information on vascular structures in the area of arthroscopic interventions is hindering the accurate predictions of procedure outcomes, therapeutic success and healing rates.

The articular structures of the stifle joint are supplied by branches of the femoral artery and main artery of the hindlimb. After the femoral artery gives rise to the lateral circumflex femoral artery that supplies the sartorius, rectus femoris, vastus medialis and vastus intermedius muscles, the saphenous artery arises from the femoral artery and leads its way between the sartorius and gracilis muscles to the medial surface of the thigh. The superficial course across the deep fascia of the pelvic limb forms distal anastomoses with the continuation of the femoral artery and represents a superficial parallel collateral supply of the distal third of the extremity. Shortly after the saphenous artery detaches from the femoral artery, the descending genicular artery originates at the distal third of the thigh. It passes to the medial aspect of the stifle joint; branches out to the sartorius, vastus medialis and adductor muscles; and supplies the joint capsule and ligaments. The last branch of the femoral artery is the caudal femoral artery, which divides into branches passing between the gastrocnemius heads shortly after. These branches may also arise separately from the main trunk of the femoral artery. The caudal femoral artery supplies musculature in the region and popliteal lymph nodes [17].

According to the definition of the *Nomina Anatomica Veterinaria* [18], the femoral artery continues as the popliteal artery from the origin of the caudal femoral artery onward. It passes between the femoral condyles and over the flexor surface of the FT joint until dividing into cranial and caudal tibial artery at a variable level. In earlier articles, the passage between the two heads of the gastrocnemius muscle or the appearance of the artery underneath the adductor muscle were also described as the origin of the popliteal artery [19,20].

Despite its relatively short distance, important branches of the popliteal artery supply muscles surrounding the stifle joint and distal limb, as well as complex structures of the stifle joint. Aa. surales enter the caudal muscles of the stifle, namely, Mm. gastrocnemius, flexor digitorum superficialis, soleus, vastus lateralis, vastus intermedius and popliteus [19].

Additional to the muscular branches, different branches of the popliteal artery serve as the origin for the majority of arteries of the stifle joint in domestic mammals [1]. The proximal lateral and medial genicular arteries, middle genicular artery, and distal lateral and medial genicular arteries may be distinguished according to the literature [21,22]. Apparently, the proximal genicular arteries may be doubled [20]. In addition, smaller meniscal branches in horses are mentioned by Updike and Diesem [23]. Together with the descending genicular artery, these articular branches of the popliteal artery build up the two main vascular networks of the stifle: rete articulare genus and rete patellae [1].

A cross-anatomical study of the stifle joint of donkeys provides a good overview of the joint and shows a corrosion sample of the popliteal artery that offers the orientation of larger branches in a representative of the family Equidae [24]. While in horses, the contribution of Updike and Diesem aimed at describing the vascular supply of the stifle joint, it failed to give detailed information and adequate visual support of the findings [23]. In addition, no relevant information was given for the meniscus supply. Thus, up to now, there has still been no exact description of equine popliteal branching in horses. This lack of information may lead to an underestimation of the potential risk of vascular trauma during surgeries in the region.

Next to the clinical interest for the vascularization pattern of stifle joint structures, research on regenerative medicine with an increased number of in vitro experiments on meniscal tissue is also in need for an exact knowledge of the blood supply of the region [25]. Therefore, this study aimed at giving a detailed description of arterial supply of the stifle joint of horses, with a special focus on meniscal branches and their supply of the cartilage.

## 2. Materials and Methods

In this study, nine cadaveric pelvic limbs (obtained from six horses; seven for the latex injection technique, two for the ink injection technique) from the anatomical collection of the University of Veterinary Medicine Vienna were used. All animals died or were euthanized for reasons unrelated to this study at the equine hospital of the University of Veterinary Medicine Vienna due to terminal diseases unrelated to the stifle joint. As the study was performed on dead horses, it did not require ethical approval. However, the animals owners’ consent to the dissection of the horses and publishing the resulting data was obtained according to the standard procedure, which was approved by the ethics and animal welfare committee of the University of Veterinary Medicine Vienna. The horses were dissected according to the “Good Scientific Practice and Ethics in Science and Research” regulation implemented at the University of Veterinary Medicine Vienna. Breed, age, sex and body weight were not considered for this study.

After disarticulation from the hip joint, the fresh limb was shortened by cutting the tibia and surrounding structures. Thereby, the size of the specimen was reduced to the thigh and proximal half of the crus with surrounding structures. Muscular tissue was removed for better visual control and slowing degradation. The arteries of the non-embalmed pelvic limb were dissected, and the popliteal artery was injected with red latex milk (latex milk, Zitzmann, Wehr, Germany; acrylic color, Marabu, Bietigheim-Bissingen, Germany) through a buttoned cannula to demonstrate the vascular architecture. Pressure by hand to the plunger of the syringe was used to force latex milk into the popliteal artery under visual control. To visualize the relevance of the descending genicular artery for the supply of the rete articulare genus, this artery was injected with blue latex milk under ligation proximal to the buttoned cannula to avoid backward flow into the femoral artery. The specimens were kept at 4 °C for two to three days to allow for curing of the latex milk and dissected afterward. Estimation of the arterial diameter was performed on latex-injected vessels. After preparation, specimens were stored in an approximately 3.6% formaldehyde solution (at 4 °C). Different stages of dissection were photographed with a digital single-lens reflex camera (Nikon, Vienna, Austria). The findings are represented in composite anatomical illustrations.

The femoral artery was exposed in the femoral canal caudal to the sartorius muscle that was only covered by the medial femoral fascia. The femoral artery, descending genicular artery and popliteal artery with all their branches were dissected as far as possible (<0.5 mm in diameter) and the branching pattern was noted. Muscles, nerves and veins were excised to ensure a maximal view of the arterial branches of interest.

For visualization of the intra-meniscal arteries, the popliteal and descending genicular artery of two stifle joints (from two horses) were injected with Indian ink. Otherwise, the procedure was similar to the one described for the latex injection. After the injection and one day at 4 °C, the menisci were dissected from the FT joint and photographed until storage in formalin (~3.6%, 4 °C). With the help of these specimens, the intrameniscal arterial architecture could be better visualized.

We aimed at a detailed description of the arterial supply of the stifle joint of horses so that vessels relevant for the supply of articular structures could be described and shown herein (Figure 1, Figure 2, Figure 3, Figure 4, Figure 5, Figure 6, Figure 7 and Figure 8, Table 1).

## 3. Results

The arterial supply of the stifle joint was assured by two networks: rete patellae and rete articulare genus. Both supported each other and were fed by branches of the femoral artery and its succession. Distinct supply zones were clearly discernible and are described in the following. Arteries and branches are described in the proximo-distal order that is also followed in the presentation in Table 1.

The **femoral artery** took its course distal in the femoral canal, bound by the sartorius, gracilis and pectineus muscles, before perforating the adductor muscle and medially detaching the **saphenous artery** at the proximal third of the thigh. The saphenous artery gave off a proximal branch that supplied the superficial medial patellar region, while the main trunk continued passing distally across the gracilis muscle. After passing the medial collateral ligament, a second branch detached to return proximal and supplied the medial patellar ligament and superficial joint capsule. Branches anastomosed with those from the proximal branch. Afterward, the saphenous artery divided into a cranial and a caudal branch at the height of semitendinosus insertion site proximal to the hock.

**Figure 1:**
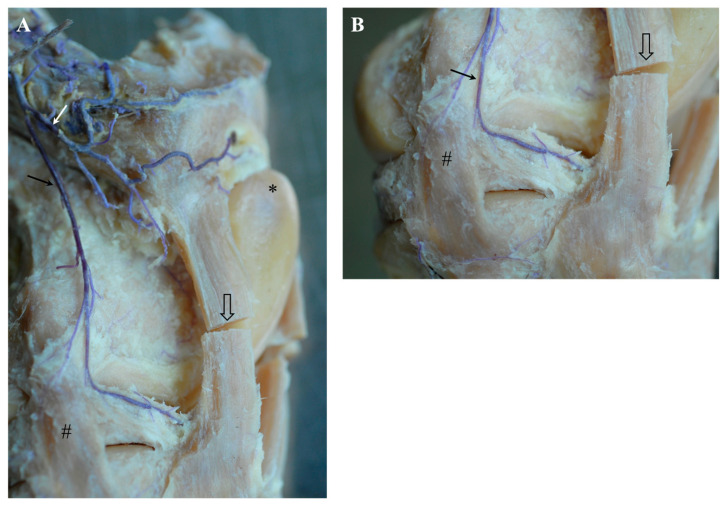
Gross dissection of left equine stifle joint demonstrating the course of the blue latex injected into the descending genicular artery, medial view. (**A**) Ramification in the patellar branch (white arrow) with numerous small branches and meniscal branch (black arrow). After releasing a small branch for the medial collateral ligament (#), the meniscal branch continued along the cranial edge of the medial collateral ligament and turned almost 90° cranially to attach to the outer aspect of the medial meniscal body. Tuberculum trochleae ossis femoris (*), cut medial patellar ligament (open arrow). (**B**) Detail of the distal course of the meniscal branch along the medial meniscus showing numerous small branches for supply of the outer aspect of the meniscus.

Shortly after the release of the saphenous artery, the ***descending genicular artery*** originated from the femoral artery in the craniodistal direction. The descending genicular artery ran—covered by the sartorius muscle—along the vastus medialis, adductor and semimembranosus muscles. It provided strong muscular branches to supply the quadriceps femoris muscle and split into two major branches to supply the articular structures, hereinafter referred to as *patellar branch* and *meniscal branch* of the descending genicular artery, respectively (Figure 1). The first or *patellar branch* of the descending genicular artery (Figure 1A: white arrow) dived through the vastus medialis muscle in the cranial direction to supply the medio-proximal region of the FP joint. The medial condyle of the femur, the medial femoropatellar ligament, and the medial parapatellar cartilaginous process and base of the patella were directly supplied by its blood (Figure 1A). Furthermore, this branch fed the rete patellae by continuing its cranial course in the direction of the patellar apex and carrying blood to the FP joint. It crossed the proximal origin of the medial patellar ligament (Figure 1A: open arrow) and passed proximal to the fat pad to the medial edge of the origin of the intermediate patellar ligament. Here, the main branch turned nearly 90° proximal to continue to supply the patella and surrounding structures. During its course, this branch of the descending genicular artery spanned a net of small vessels across the patella and participated in feeding the rete patellae.

The main branch or *meniscal branch* of the descending genicular artery (Figure 1A: black arrow) headed craniodistally in the direction of the origin of the medial collateral ligament (Figure 1: #), giving rise to additional small muscular branches and continuing at the cranial aspect of the medial collateral ligament (Figure 1A,B). The medial side of the distal part of the femur, like the trochlea, condyle and epicondyle, as well as the medial collateral and medial patellar ligaments, were supplied along its course. The continuation of the descending genicular artery passed the FT joint space and attached between the medial collateral ligament and the medial patellar ligament to the cranial third of the lateral articular meniscus (Figure 1A,B). Small accessory branches were released to the articular meniscus while it coursed along its lateral side up to its cranial horn (Figure 1B: arrow heads). After also supplying the ligamentum tibiale craniale menisci mediale, there were numerous anastomoses with branches of the middle genicular artery. Next to these, small branches of the descending genicular artery descended between the medial and intermediate patellar ligament distal and finally ramified within the patellar ligaments’ insertions and the tibial periost.

The origin of the ***caudal femoral artery*** near the two heads of the gastrocnemius muscle marked the transition of the femoral to popliteal arteries. The caudal femoral artery divided shortly after being released from the femoral artery in two main branches, which supplied muscles of the thigh region, like the gastrocnemius, flexor digitorum superficialis, biceps femoris, vastus lateralis, adductor, semimembranosus and semitendinosus muscles. In all but one leg examined, the branches arose separately from the femoral artery.

### Popliteal Artery

The continuation of the femoral artery beyond the origin of the caudal femoral artery is termed the popliteal artery. With up to 1.3 cm, the largest artery of the region passed between the heads of the gastrocnemius muscle and ran medial to the superficial digital flexor muscle over the caudal face of the femur and then over the flexor surface of the FT joint capsule while being covered by the popliteal muscle. The popliteal artery gave rise to several branches on its course through the popliteal notch before heading lateral and being divided into cranial and caudal tibial arteries at the interosseous space between the tibia and fibula.

**Figure 2:**
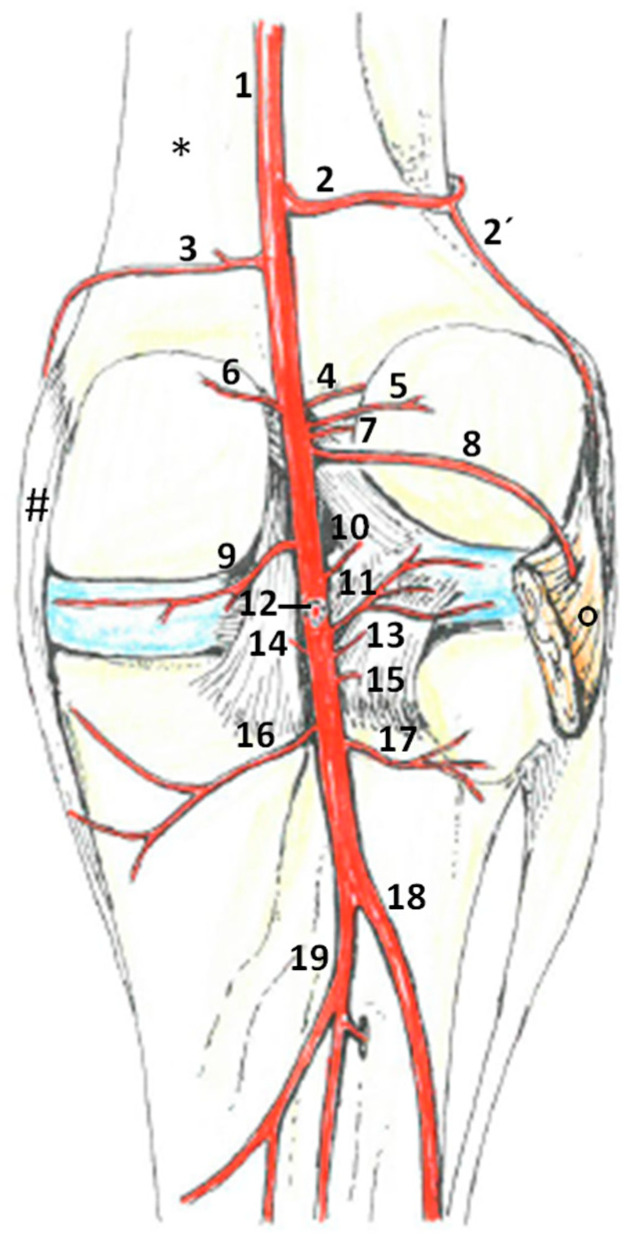
Anatomical drawing of the arterial blood supply in the right popliteal region of an equine stifle joint. Articulating femur (*) and tibia, with menisci in light blue, were stabilized by several ligamentous structures (e.g., medial collateral ligament #) and muscles (here: popliteal muscle °). Branches of the popliteal artery that were relevant for stifle joint structures are listed in proximo-distal order: 1—popliteal artery, 2—proximal lateral genicular artery, 2’—meniscal branch, 3—proximal medial genicular artery, 4—proximal cranial genicular artery, 5—lateral capsular branch, 6—medial capsular branch, 7—proximal ligamental branch, 8—popliteal muscle branch, 9—medial meniscal branch, 10—middle ligamental branch, 11—lateral meniscal branch, 12—middle genicular artery, 13—accessory lateral meniscal branch, 14—distal medial ligamental branch, 15—distal lateral ligamental branch, 16—distal medial genicular artery, 17—distal lateral genicular artery, 18—cranial tibial artery and 19—caudal tibial artery. Next to the generally described middle genicular artery (12), as well as two proximal and distal genicular arteries (2, 3 and 16, 17), there were a multitude of regularly occurring branches comparable in lumen that supplied the stifle joints of the horses shown here and described in the text.

The popliteal artery gave rise to several branches, which are described in the following from proximal to distal in their order of origin (Figure 2, Table 1).

**Figure 3:**
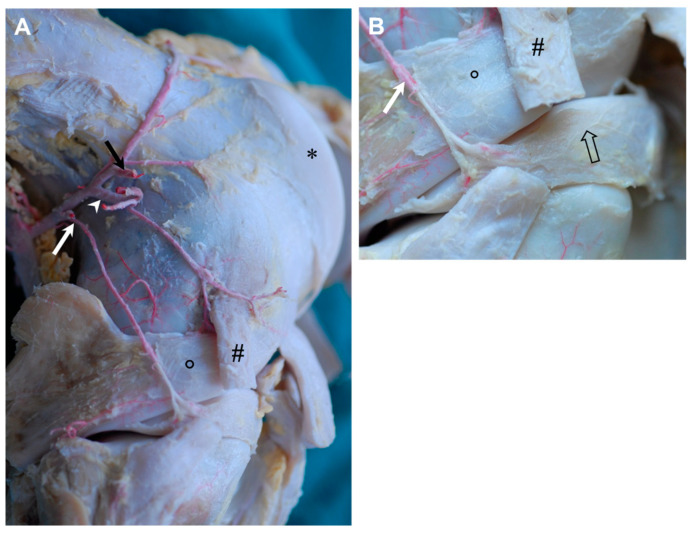
Gross dissection of the right equine stifle joint demonstrating the ramification of the red latex injected into the proximal lateral genicular artery, lateral view. (**A**) Overview of the course of the three main branches of the proximal lateral genicular artery. The meniscal branch (white arrow), distal branch (white arrowhead) and patellar branch (black arrow). After releasing small branches for the lateral epicondyle, the meniscal branch continued across the tendon of the popliteal muscle (open circle) and attached to the outer aspect of the lateral meniscal body, femoral trochlea (*). (**B**) Detail of the meniscal branch (white arrow) and its attachment to the body of the lateral meniscus. Latex did not fill the continuation of meniscal branch; therefore, the vessel is seen in white here (open arrow). Cut lateral collateral ligament (#).

The ***proximal lateral genicular artery*** was released as the first branch of the popliteal artery at the proximal aspect of the lateral condyle of the femur. Smaller branches were given off to the surrounding muscles and the joint capsule pouched proximally under the lateral head of the gastrocnemius muscle. Three main branches could be distinguished (Figure 3): A *meniscal branch* (Figure 3A,B: white arrow), which left in the craniodistal direction, passed the tendon of the popliteal muscle and attached to the outer aspect of the lateral meniscal body right before diving below the lateral collateral ligament (Figure 3A,B: #). A *distal branch* approximately 2 mm in diameter (Figure 3A: white arrowhead) supplied the musculature, like the vastus lateralis, biceps femoris and semitendinosus muscles, before it was partially attached to the fibrous joint capsule. It ran across the joint capsule in the lateral direction and turned to the lateral collateral ligament (Figure 3A: #) that was also supplied by this branch in its region of origin. The third branch—the *patellar branch* (Figure 3A: black arrow)—ran proximolateral to supply the apex patellae, as well as the joint capsule around the insertion of the vastus lateralis muscle.

Slightly distally to the proximal lateral genicular artery, at the proximal margin of the popliteal muscle, the ***proximal medial genicular artery was*** released by the popliteal artery. It ran medial over the joint capsule, covered by the medial head of the gastrocnemius muscle, and terminated in the region of the medial collateral ligament.

**Figure 4:**
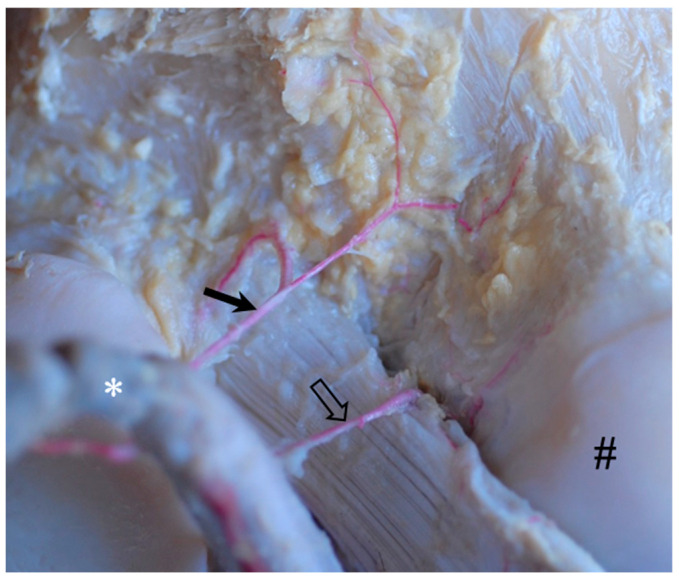
Proximo-caudal aspect of the right equine stifle joint with the dissected popliteal artery drawn caudal and slightly medial to allow for a view to the insertion of the meniscofemoral ligament at the popliteal surface of the femur. Detail of the latex injected popliteal artery (white *) with its proximal cranial genicular branch (black arrow) to the popliteal surface of the femur. Further distal, there was an additional cranial branch of the popliteal artery (proximal ligamental branch, open arrow) passing over the lateral edge of the meniscofemoral ligament to supply deeper aspects of the ligament and femur. Lateral condyle of the femur (#).

The next branch of the popliteal artery ran cranial in the direction of the popliteal surface to be divided into a *proximal cranial genicular branch* (Figure 4: black arrow) supplying the proximal aspects of the meniscofemoral ligament and another small branch (Figure 3: open arrow) carrying blood to the distal end of the femur (Figure 4). Both branches had separate origins in 44% of cases (four animals).

**Figure 5:**
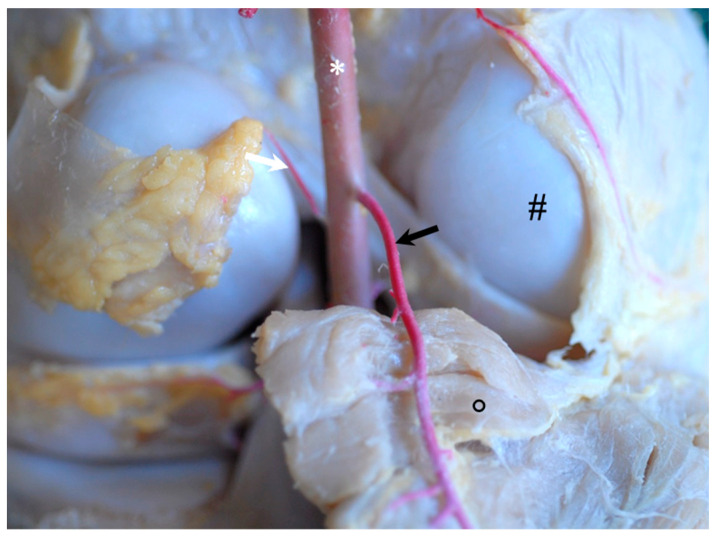
Caudal aspect of a right equine stifle joint with detail of the red latex injected popliteal artery (white *). Caudal branch of the popliteal artery (popliteal muscle branch) supplying the popliteal muscle (black arrow to the branch, open circle on the popliteal muscle). The medial capsular branch is also visible in the background (white arrow) and transferred blood to the joint capsule (partly removed). Lateral condyle of the femur (#).

Two branches with a diameter less than one millimeter left the popliteal artery near the proximal half of the femoral condyles to supply the caudal aspect of the stifle joint capsule. The proximal branch headed to the lateral side (*lateral capsular branch*, Figure 5: without description) and the distal branch to the medial aspect of the capsule (*medial capsular branch*, Figure 5: white arrow). These vessels were found in five animals each (56%). In one case, the *medial capsular branch* also supplied the popliteal muscle.

In all but one animal (89%), another small branch left the popliteal artery at the mid-height of the femoral condyles into the cranio-proximo-lateral direction to supply the meniscofemoral ligament (*proximal ligamental branch*, Figure 4: open arrow).

The subsequent vessel left the popliteal artery in a caudal direction and had a diameter of about 3–4 mm. This branch descended over the proximal margin of the popliteal muscle to enter the middle of the muscle belly (*popliteal muscle branch*, Figure 5: black arrow). In one animal, this vessel gave rise to a branch supplying the lateral meniscus, and in another, the lateral part of the articular capsule.

**Figure 6:**
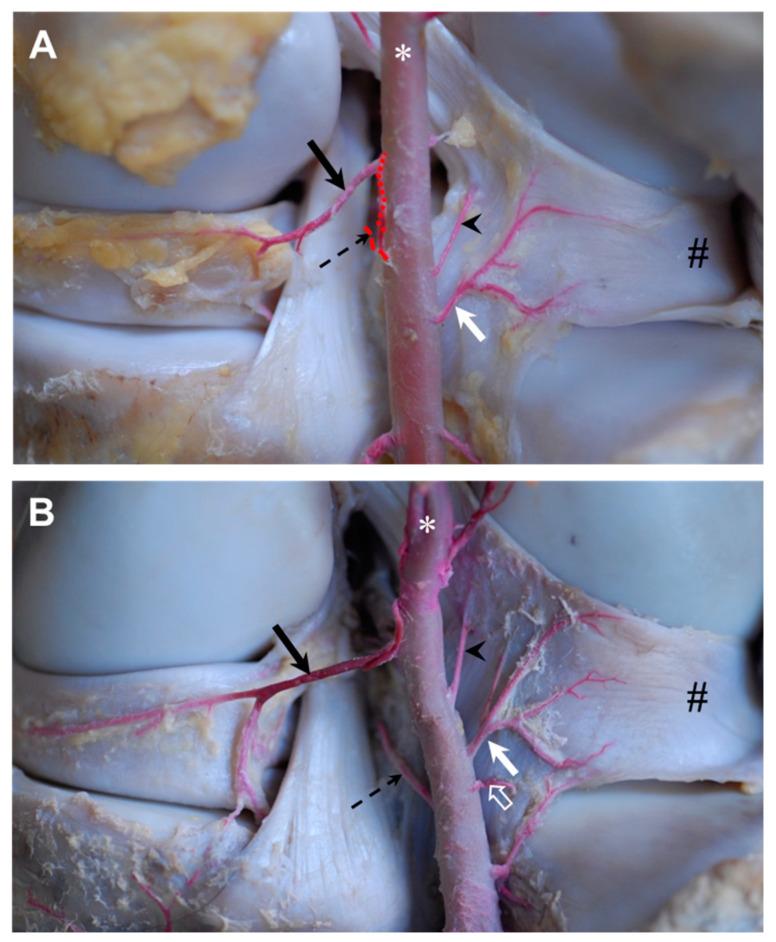
Caudal aspect of right stifle joints of two horses with a latex-injected popliteal artery (*) and its meniscal branches. The medial meniscal branch (black arrow) left the popliteal artery proximal to the medial meniscus, crossed the caudal cruciate ligament and coursed along the outer aspect of the meniscus, supported by several very small branches. The lateral meniscal branch (white arrow) left the popliteal artery only shortly before reaching the distal edge of the lateral meniscus, near the caudal ligament of the lateral meniscus (#). Proximal to the lateral meniscal branch, a small vessel was constantly found that passed from the popliteal artery to the angle between the meniscofemoral ligament and the caudal ligament of the lateral meniscus (middle ligamental branch, arrowhead). In (**A**), the lateral meniscal branch (white arrow) is ramified in two main branches, with one supplying the proximal aspects and the other supplying the distal aspect of the caudal horn of the lateral meniscus. In (**B**), the lateral meniscal branch (white arrow) is ramified in one very small branch heading to the base of the meniscofemoral ligament and three branches for the proximal, middle and distal aspect of the lateral meniscus. Just distal to the lateral meniscal branch, there was an accessory lateral meniscal branch of the popliteal artery supporting the lateral meniscal branch in its distal supply zone (white open arrow). The branch running to the inner aspect of the caudal cruciate ligament (distal medial ligamental branch, black dashed arrow) left the popliteal artery at variable heights. In (**A**), the distal medial ligamental branch left the popliteal artery at the height of the middle ligamental branch (actual course of the branch is visualized by a red dashed line, while the red dotted line shows the branch after it was cut and bent proximal along the popliteal artery), while in (**B**), it is larger in diameter and left the popliteal artery only at the height of the accessory lateral meniscal branch.

The branch to the medial meniscus (medial meniscal branch, Figure 6) had a diameter of about one millimeter and came into contact with the meniscus shortly after having passed the caudal cruciate ligament. The main meniscal branch continued along the midline of the meniscus and was visible until the medial margin of the medial femoral condyle was reached. During its course, several very small branches left the vessel to supply the medial meniscus and the joint capsule. The supply to the caudal horn of the meniscus was ensured either by a first branch (33%, three animals) or by an additional small meniscal branch from the popliteal artery directly (67%, six animals).

**Figure 7:**
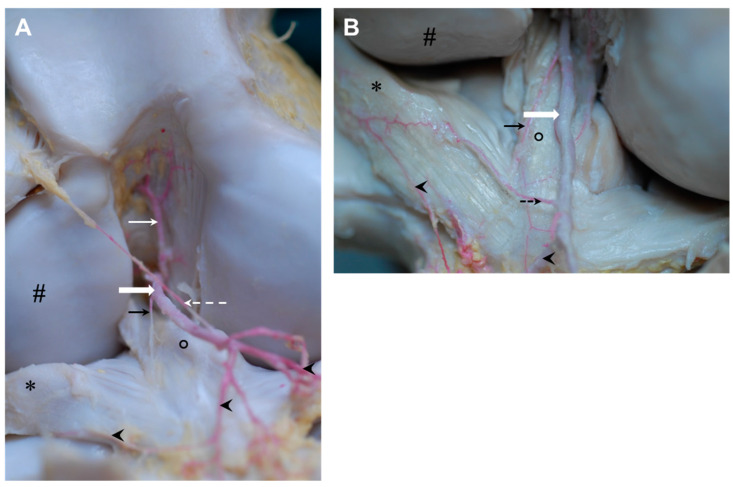
Cranial (**A**) and proximocranial (**B**) views of a maximally flexed right equine stifle joint after transection of patellar ligaments and translocation of the patella. Middle genicular artery (bold white arrow) and its branches were injected with red latex via the popliteal artery. First, a branch to the caudal cruciate ligament (white arrow) left the middle genicular artery that also supplied the tissues between the condyles of the femur (not visible in (**B**)). The middle genicular artery passed on top of the cranial cruciate ligament (open circle) to the cranial aspect of the stifle joint, releasing a first small branch (black arrow) that supplied the cranial cruciate ligament, as well as the inner aspects of the cranial horn of the lateral meniscus (*). It was supported by a variably occurring cranial branch (black dotted arrow in (**B**)) leaving the middle genicular artery at the area of the cranial meniscal ligaments; several small branches left the middle genicular artery in a proximal direction to supply the fat pad and capsular structures (white dotted arrow in (**A**)). The main ramification occurred at the cranial intercondylar area giving rise to several branches for the fat pad, patellar ligaments and tibia. Here, two additional meniscal branches to the outer aspects of the cranial horn of both menisci also left the middle genicular artery (arrow heads). Lateral condyle of the femur (#).

The ***middle genicular artery*** was about five millimeters in diameter and the largest branch of the popliteal artery (Figure 7: bold white arrow). In one case, the middle genicular artery was the origin for the *medial meniscal branch*, instead of the latter arising from the popliteal artery; however, the branching occurred within 5 mm. The middle genicular artery headed cranially through the intercondylar fossa of the femur and between the cruciate ligaments to divide into branches for the latter, the infrapatellar fat pad and synovial membrane, cranial aspects of the menisci and their cranial attachment, patellar ligaments and surrounding structures. The following meniscal branches of the middle genicular artery could be differentiated: (i) a small branch carrying blood to the inner aspect of the cranial horn of the lateral meniscus (Figure 7: black arrow); (ii + iii) one small branch each to the outer aspect of the cranial horn of the lateral and medial meniscus, respectively (Figure 7: arrow heads); and (iv) an additional small branch heading further along the outer circumference of the cranial horn of the medial meniscus until the area of the medial patellar ligament was reached.

Anastomoses with branches of the descending genicular artery were observed in the fat pad, as well as with the branches for the outer aspect of the cranial horn of the medial meniscus (iii and iv).

In one case, the middle genicular artery was located further distally so that the *medial meniscal branch* ended proximal to it.

In all but one stifle, the small branch to the angle between the distal aspect of the meniscofemoral ligament and the ligamentum tibiale caudale menisci lateralis was present (89%, all but one animal). Both ligamentous structures were supplied by this *middle ligamental branch* (Figure 6: arrowhead).

The branch to the lateral meniscus (*lateral meniscal branch*, Figure 6: white arrow) originated from the popliteal artery and attached to the lateral meniscus directly lateral to the ligamentum tibiale caudale menisci lateralis, which was also supplied. There were two main branches for the meniscus continuing along its proximal and distal margins (Figure 6A: white arrow). Sometimes they were supported by a third branch travelling in the midline (Figure 6B: white arrow). Some sub-branches also supplied the caudal meniscal ligament. In three cases, there was an *accessory lateral meniscal branch* present (33%) fed by the popliteal artery either originating proximal (22%, two animals) or distal (11%, one animal) to the main *lateral meniscal branch* (Figure 6: open white arrow).

A very small branch (*distal medial ligamental branch*, approximately 1 mm, Figure 6: dashed black arrow) headed straight along the inner aspect of the caudal cruciate ligament. In one of the stifles, this vessel originated from the *medial meniscal branch* instead of coming from the popliteal artery directly.

Distal to the level of the proximal articular surface of the tibia, a very small vessel left the popliteal artery to supply the distal attachment of the ligamentum tibiale caudale menisci lateralis at the area intercondylaris caudalis (*distal lateral ligamental branch*). It had a diameter of less than 1 mm and was supported by collaterals (44%, four animals) and/or left a branch to the lateral meniscus (22%, two animals, Figure 6B).

The ***distal medial genicular artery*** left the popliteal artery distal to the tibial condyle. The distal medial genicular artery headed straight medially along the bone to be divided into a *proximal branch* to the medial tibial condyle and joint capsule and a *distal branch* leading blood to the region of the lateral collateral ligament attachment. There were anastomoses between the proximal branches and branches of the medial meniscal branch that continued to the articular capsule, as well as with distal aspects of the meniscal branch of the descending genicular artery. The situation was similar for all specimens under study.

The ***distal lateral genicular artery*** could be found distal to the distal medial genicular artery. It passed along the caudal tibia surface and diverted in branches heading proximal to the lateral tibial condyle and supplying the articular capsule, as well as heading distal to the fibula head with additional branches to the deep digital flexor muscle in all specimens.

Shortly after, the lateral distal genicular artery left the popliteal artery, some tiny muscular branches were given off, and the popliteal artery split into cranial and caudal tibial arteries.

**Figure 8:**
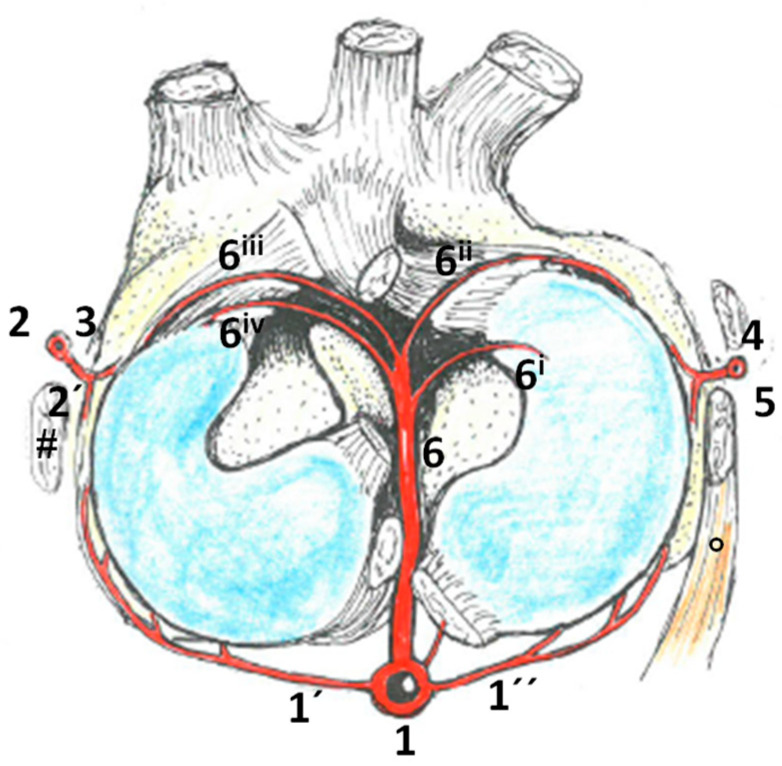
Schematic visualization of the vascular supply of equine menisci. Top view of the right tibial plateau with menisci (in light blue) and ligamental structures (e.g., # medial collateral ligament); the femur and surrounding muscles were removed for better visual perception (except for the popliteal muscle (°)). Branches of the popliteal artery (1) and descending genicular artery (2) provide the main supply for the meniscal tissue. The rete articulare genus and rete patellae could also be fed by branches of the saphenous artery, which, however, did not play a crucial role in the meniscal vascularization. Caudal horns of the menisci were directly supplied by medial (1’) and lateral (1’’) meniscal branches of the popliteal artery. The latter could be supported by accessory lateral meniscal branches. The medial meniscal body was supplied by the meniscal branch of the descending genicular artery (2’), which attached to the outer aspect of the meniscus right cranial of the medial collateral ligament. Blood of the proximal branch of the distal lateral genicular artery (3) supplied the joint capsule and therewith also the meniscal tissue; however, this was only to a smaller degree. The lateral meniscal body was supplied by blood from the meniscal branch of the proximal lateral genicular artery (4) that passed over the tendon of the popliteal muscle and attached the meniscus just caudal of the lateral collateral ligament. In addition, anastomoses were found in this area with meniscal branches of the distal lateral genicular artery (5) connecting to the outer aspects of the joint capsule and meniscus cranial to the lateral collateral ligament. The cranial horns of the menisci were supplied by meniscal branches of the middle genicular artery (6). While the lateral meniscus was supplied on both the inner and outer aspects of its cranial horn (6i,6ii), the medial meniscus was supplied by two branches both delivering blood to the outer aspects of the cranial horn (6iii,6iv).

The vascularization of the menisci was ensured by branches of the popliteal artery, as well as the meniscal branch of the descending genicular artery (Figure 8). The caudal horns were fed by medial and lateral meniscal branches of the popliteal artery. While the medial meniscal branch was long and reached until the medial collateral ligament, the lateral meniscal branch was more ramified and often supported by accessory lateral meniscal branches; however, it only visibly reached half of the caudal horn. The cranial horns of the menisci were supplied by branches of the middle genicular artery that were ramified in the area of cranial intercondylar fossa. The cranial horn of the lateral meniscus was directly supplied at its inner and outer sides, while branches for the cranial horn of the medial meniscus were only visible at the outer aspects. The bodies of the menisci were supplied from two directions: proximal from the descending genicular artery on the medial side (Figure 1 and Figure 7) and the proximal lateral genicular artery on the lateral side. Both branches attached to the outer aspects of the meniscus right cranial to the collateral ligaments. The distal vascular supply was less obvious, and the arteries were smaller; however, on both sides, anastomoses with the proximal branches of the medial/lateral distal genicular artery, respectively, were visible.

Branches of the popliteal artery that were relevant for the menisci are listed:

1—popliteal artery, 1’—medial meniscal branch, 1’’—lateral meniscal branch, 2—descending genicular artery, 2’—meniscal branch, 3—proximal branch of the distal medial genicular artery, 4—meniscal branch of the proximal lateral genicular artery, 5—proximal branch of the distal lateral genicular artery, 6—middle genicular artery, 6i—branch to the inner aspect of the cranial horn of the lateral meniscus, 6ii/iii—branches to the outer aspect of the cranial horn of the lateral/medial meniscus and 6iv—accessory branch to the outer aspect of the cranial horn of the medial meniscus.

## 4. Discussion

Blood supply to the equine stifle joint is guaranteed by branches of the saphenous artery, descending genicular artery and numerous regularly occurring branches of the popliteal artery. Next to the two well-known proximal genicular, two distal genicular and one middle genicular artery deriving from the popliteal artery [21,22], this article describes eleven additional branches that generally and consistently occur in horses. Despite their small diameters, they seemed to play a crucial role in direct blood supply to articular structures, as well as additional supply of the rete articulare genus. For clarity, the discussion of the findings is presented in the same proximo-distal order of vascular branches as the results.

In addition, for potential importance in regenerative medicine, the vascular pattern of equine menisci is presented in detail, with all relevant sources of blood supply in an overview.

The vascular pattern differed slightly between individuals. Variability especially occurred in the total number of small vessels, whereas the area of supply of a certain artery was usually maintained. Variable occurrence is widely known for venous structures, but also arteries show inter-individual differences, where even large vessels like the terminal branching of the popliteal artery in the lower limb of human or equine show varieties [26,27,28]. The effects of breed, age, sex and body weight could not be evaluated in this study due to missing information, the limited sample size and the different scope of the study. Future studies could include this information to evaluate potential statistically significant influencing factors.

In horses, the proximal branch of the saphenous artery supplies the medial patellar region and capsule of the FP joint. This branch feeds into the rete patellae too. This finding is in accordance with information from Updike and Diesem, who had already described saphenous artery involvement in stifle joint vascularization of horses in 1980 [23].

The descending genicular artery gave off two branches to supply the stifle joint: the patellar branch for vascularization of the FP joint and a meniscal branch to the medial meniscus and surrounding structures of the FT joint (see Figure 1). Updike and Diesem described different branches of the descending genicular artery: a muscular branch for vascularization of the vastus medialis muscle that corresponded well with our findings of strong muscular branches to the quadriceps muscle and a cranial branch whose area of distribution corresponded with what we termed the patellar branch of the descending genicular artery [23]. The later ramification in a proximal and distal branch, with both supplying the patellar region described by Updike and Diesem, could not be confirmed by our results, as we found varying subsequent courses and diameters of branches [23].

We were not able to relate information about the two additional branches (deep and distal branches) described by Updike and Diesem to the situation found in our specimens [23]. The supply of the medial meniscus´s body was obvious and consistently found in all of the animals in our study (see Figure 1B) but not described in Updike and Diesem [23]. A possible explanation for this discrepancy might be the inconsistent description by Updike and Diesem, as the explanation of ramification of the descending genicular artery differs between the main text and the related illustration in the article [23]. Moreover, the level of detail of the figure is insufficient such that reliable information is limited. In donkeys, arterial blood supply was found to originate from the descending genicular artery, the caudal femoral, popliteal and cranial tibial arteries [24]. Unfortunately, no further ramification is mentioned. While the participation of the cranial tibial artery is also known for dogs [1], we could not verify this situation for horses and are, therefore, in accordance with Ghoshal and Getty [19].

In humans, the descending genicular artery seems to be regularly involved in the vascularization of the stifle joint. The according branches are termed the articular branch or supreme genicular artery, as it is the most proximal artery to feed the peripatellar vascular anastomotic ring [29,30]. As the patellar and meniscal branches of the descending genicular artery in our study were regularly occurring and stable in their course and area of supply, we are confident to assume generality for horses.

Even though not relevant for joint vascularization, the origin of the caudal femoral artery is of interest here as it demarcates the renaming of the femoral artery to the popliteal artery. Even though in other domestic species, several collaterals are known and the last one was named the distal caudal femoral artery to define the start of popliteal artery, in horses, this differentiation is not used in the official veterinary anatomical nomenclature [21]. Of the nine stifle joints analyzed for our study, only one specimen showed a single caudal femoral artery (which divided into two main branches shortly after having left the femoral artery). The nomenclature of the caudal femoral artery to the proximal and distal caudal femoral arteries could be considered to mirror the situation in situ and homogenize nomenclature within domestic animals.

The proximal lateral and proximal medial genicular arteries are well described and our findings confirm the existing literature [1,20,23]. The superior lateral genicular artery of humans that corresponds to the proximal lateral genicular artery in horses is not involved in human articular vascularization [31], whereas in horses, it feeds the rete patellae and supplies the joint capsule and adjacent structures directly.

Proximal to the middle genicular artery, six constantly occurring vessels left the popliteal artery to supply joint related structures of the stifle. Other than a capsular branch from the popliteal artery described by Benedetto et al. [31], no other branches of the popliteal artery have been sufficiently described. This may be due to the fact that preparation in the popliteal region is quite difficult, especially when it comes to very delicate structures, like small vessels. The genicular fascia is a compact and strong connective tissue sheet such that during preparation, small vessels might tear and dissemble a vessel-free situation. Moreover, the small arterial diameters assessed in our study might be an overestimation, as the injection of latex may expand the vessels. Despite their small lumina, we are confident to add importance to these small vessels. Their regular occurrence reflects a constant and general need for blood supply to the joint structures analyzed. The net formation itself suggests the necessity of thorough vascularization and underpins the importance of afferent vessels. The rete patellae and rete articulare genus could not be visualized completely due to the limitations of the latex technique. For full visualization, one would need to apply a medium with high fluidity and low surface tension (like Methacrylat for SEM); computed tomography after injection of a contrast medium and digital three-dimensional modelling is another appropriate possibility, although even then, a functional closure of some arteries might prevent the complete visualization of the vascular tree. However, our goal was to analyze the main arterial branches supplying the stifle joint, and thus, the latex technique was sufficient in this respect.

The aim of the emerging field of regenerative medicine is to engineer cell or tissue replacements for human tissue that faces a high risk of injury or wearout. The meniscus is an example of human tissue that is often affected by both injury and wearout [32], and great effort has been made to find ways to replace destroyed tissue and restore stifle joint function. Identifying appropriate animal models that reflect the situation in humans is the first step to success in regenerative medicine research. Showing similar pathologies in the region of interest, namely, meniscus injury, but also dysfunction as a consequence of wearout, a horse is one of the few animals that seems to be suitable in this respect [33] and would also benefit from the results as a patient in the scope of translational medicine. Next to the comparable meniscal pathologies, structural properties also need to be comparable to that of humans for equine meniscus to serve as adequate research tissue in regenerative medicine. While the general anatomical characteristics have been described in veterinary anatomical textbooks for a long time [1,34], histological and biomechanical properties have only been characterized in the last decade [35,36,37].

The first pilot studies in equine meniscus tissue engineering emerged [38] and posed the question regarding physiological blood supply, as this is crucial for the regeneration and repair of tissue and, therefore, also essential for studies on tissue engineering.

Even though the general arterial blood supply is known from textbooks, there have been only very few attempts to describe the fine structures of equine meniscal branches of the popliteal artery [20,23]. Both studies lacked a precise description and were inconsistent in their results. Therefore, the detailed description and display of meniscal branches of the popliteal artery and especially the vascularization pattern of equine menisci in the article at hand is relevant and needed.

The middle genicular artery showed the largest diameter of vessels branching off the popliteal artery. This finding is in accordance with measurements of König, who stated about 2.1 mm as the diameter of the middle genicular artery versus 0.1–1.0 mm for the proximal and distal genicular arteries [20]. According to our findings, the middle genicular artery supplied the crucial ligaments, fat pad, joint capsule and menisci. Arnoczky and Scapinelli [39,40,41] found a similar field of vascularization in both dogs and humans, where the middle genicular artery supplies the anterior cruciate ligament, fat pad and surrounding structures with blood. Four meniscal branches supplying the cranial horns of the menisci could be distinguished (see Figure 6): one vascularizing the inner circumference of the cranial horn of the lateral meniscus, one that supplies the outer circumference of the cranial horn on each side and an additional vessel carrying blood further along the outer circumference of the cranial horn of the medial meniscus. This finding is, in general, comparable with the situation in humans, as Benedetto et al. also described the middle genicular artery to release branches to the cranial horns of human menisci [31]. However, our findings are in contrast to the description of Updike and Diesem, who did not mention any branches to the cranial horns of the menisci [23]. However, they described a small branch to the caudal aspect of the medial meniscus, which is also found in humans (Benedetto et al., 1985) [31]. As we did not see any branches of the middle genicular artery prior to passing the cruciate ligaments, except in one case where the medial meniscal branch of the popliteal artery did not leave the mother vessel directly but shared 5 mm with the middle genicular artery, this might be an explanation for the described situation in Updike and Diesem in horses [23]. It was not mentioned in how many cases they could verify this situation. Also, the order of vessels leaving the popliteal artery was not unambiguously described in Updike and Diesem, even though they tried to describe landmarks for orientation [23]. We are skeptical of using landmarks, as during joint movement, these landmarks and distances between them can change rapidly. We favor a description per order of appearance and give additional information about the numbers of cases and potential alternatives or collaterals. Arteries leaving the popliteal artery proximal to the distal genicular arteries are, to our knowledge, described in detail for the first time in this paper. The small lateral and medial branches with lumen of about 1 mm seem to be important, especially for ligamentous structures in the region.

The distal medial genicular artery constantly leaves the popliteal artery proximal to the distal lateral genicular artery, which is in contrast to the order of appearance in the description of Updike and Diesem and the *Nomina Anatomica Veterinaria* [18,23]. The order of the proximal genicular arteries is in accordance with the relevant literature. The distal medial genicular artery ramifies in a proximal and a distal branch, with both being involved in vascularization of the joint capsule, collateral ligament and adjacent structures. Anastomoses with the medial meniscal branch and meniscal branch of the descending genicular artery were observed, emphasizing their importance for the supply of the rete articulare genus (see Figure 6).

In general, our findings show branches of the popliteal artery supplying the stifle joint, which were very small in caliber, in many cases less than 1 mm. Nevertheless, we suspect we overestimated the diameters, as the injection of latex material into ligated arteries may result in expansion of the vessel lumen. We observed that not all branches were filled; therefore, we might have overlooked further small branches during preparation or might have ripped or cut small vessels during preparation. The preparation of delicate vascular structures proved to be difficult, especially in the popliteal region, where the articular capsule and surrounding fibrous structures are very firm.

Up to now, a detailed and systematic description of vascularization to equine menisci has been missing. Based on our analyses, we were able to describe the blood supply of the individual parts of the menisci (see Figure 6). Especially for surgical interventions [42,43] or diagnostic arthroscopy [16] in horses, this information is crucial to avoid harm to vessels and dependent structures in the area of action. The popliteal artery is large in diameter and its injury can be avoided quite easily. However, it is the smaller vessels that are at risk of being overseen or their function of being underestimated. Even though there may be collaterals and the genicular network to maintain general vascularization, our findings clearly show the constantly occurring vessels that are obviously needed for the local supply of blood, especially in cases of articular structures, like the capsule, ligaments or menisci, that play a crucial role in stifle joint function and equine locomotion. Cranial approaches to caudal aspects of the FT joint seem to be especially risky in our view, as the arthroscope is passed through the intercondylar space along the caudal cruciate ligament [15], exactly where the middle genicular artery is located, which is the main vessel for vascular supply of the cranial horns of the menisci. The risk to damage the popliteal artery is stated for almost all arthroscopic approaches; however, smaller branches, even those that are at a higher risk to be damaged, are not mentioned at all [13,14,15]. This might be due to the fact that these vessels have not been described in detail yet. For humans, de Carvalho et al. pointed out the risk of damaging the middle genicular artery as a result of injuries or during surgical or arthroscopic interventions [44]. According to these authors, the underestimation of vascular damage as a surgical complication is due to the lack of anatomical knowledge, which is a statement that applies to equine medicine too [44].

Considering the vascularization of the menisci, we found three points for larger vessels to attach to the meniscus: from the caudal side to the caudal horn, from the proximal and distal side to the body of the menisci just cranial to the collateral ligaments, and from the cranial side to the cranial horn (see Figure 6). The body of the meniscus is mentioned to be less vascularized than the horns in humans [31]. We could not verify or disprove this statement for horses, as we did not perform any kind of quantification. Concerning the diameter of the relevant vessels, we could not see any differences between the locations. What was, however, obvious was that there were two areas where the blood supply was limited: first the cranial aspect of the caudal lateral horn, as the popliteal tendon prevented direct vascular supply in this area, and second, the inner aspects of the medial cranial horn, as there was no visible direct supply by branches of the middle genicular or any other artery. This is in contrast to the lateral meniscus, where branches feed both the inner and outer circumferences of the meniscus. Interestingly, the medial horn is especially prone to lesions, as Davis et al. (2022) found over 82% of defects there [45]. Even more specifically, the medial cranial horn was reported with 79% involvement of this area in stifle-injured horses by Walmsley [46]. The high prevalence is brought in connection with a combination of greater overall cranial-caudal translocation, along with the least mobility in the cranial horn of the medial meniscus in horses [47]. Limited vascular supply might add to this susceptibility to mechanical strain and even impair repair actions.

## 5. Conclusions

Numerous consistent and generally occurring arterial branches of the femoral and popliteal arteries are described and presented and expand the knowledge of equine stifle joint vascularization. In both diagnostic and therapeutic interventions of complex structures, like the stifle joint, the protection of pivotal arterial blood supply should have the highest priority. Knowledge of the ramification and supply of delicate structures is essential in limiting the damage and long-term impairment of joint structures to a minimum. The newly generated knowledge on vascularization pattern in the stifle joint region will help equine surgeons choose the best approach and more accurately assess potential complications.

In addition, the map of meniscal supply is potentially of great importance to both clinicians in preparation for the best-suited incisions for arthroscopy, as well as for researchers designing new approaches on meniscal studies and choosing suitable animal models.

## Figures and Tables

**Table 1:** Overview of the ramification of femoral and popliteal arteries relevant to the stifle joint. Vessels already described in *Nomina Anatomica Veterinaria* [18] are listed in bold. The caudal femoral artery marks the transition from the femoral to popliteal artery but is not relevant for the vascularization of stifle joint structures; therefore, it is put in brackets. Newly described additional branches, as well as further ramification, is shown in italics.

Main Artery	First-Order Ramification	Further Ramification with Relevance to the Stifle Joint
**Femoral artery**	***Descending genicular artery***	*Patellar branch*
		*Meniscal branch*
	(***Caudal femoral artery***)	
**Popliteal artery**	***Proximal lateral genicular artery***	*Meniscal branch*
		*Distal branch*
		*Patellar branch*
	***Proximal medial genicular artery***	
	*Proximal cranial capsular branch*	
	*Proximal lateral capsular branch*	
	*Proximal medial capsular branch*	
	*Proximal ligamental branch*	
	*Popliteal muscle branch*	
	*Medial meniscal branch*	
	***Middle genicular artery***	*Inner cranial lateral branch*
		*Outer cranial lateral branch*
		*Outer cranial medial branch*
		*Accessory outer cranial medial branch*
	*Middle ligamental branch*	
	*Lateral meniscal branch*	*Proximal branch*
		*Distal branch*
	*Accessory lateral meniscal branch*	
	*Distal medial ligamental branch*	
	*Distal lateral ligamental branch*	
	***Distal medial genicular artery***	*Proximal branch*
		*Distal branch*
	***Distal lateral genicular artery***	

## Data Availability

Data are available at the University of Veterinary Medicine Vienna.
